# Ethyl 2,6-dichloro-4-phenyl­quinoline-3-carboxyl­ate

**DOI:** 10.1107/S1600536809045334

**Published:** 2009-11-04

**Authors:** S. Mohana Roopan, F. Nawaz Khan, M. Vijetha, Venkatesha R. Hathwar, Seik Weng Ng

**Affiliations:** a Chemistry Division, School of Science and Humanities, VIT University, Vellore 632 014, Tamil Nadu, India; b Solid State and Structural Chemistry Unit, Indian Institute of Science, Bangalore 560 012, Karnataka, India; c Department of Chemistry, University of Malaya, 50603 Kuala Lumpur, Malaysia

## Abstract

In the title compound, C_18_H_13_Cl_2_NO_2_, the quinoline ring system is almost planar (r.m.s. deviation 0.009 Å), and the phenyl and carboxyl­ate planes are twisted away from it by 59.2 (1) and 65.9 (2)°, respectively.

## Related literature

The title compound is a 6-chloro analouge of ethyl 2-chloro-4-phenyl­quinoline-3-carboxyl­ate, which has been examined for endothelin binding affinity; for details, see: Anzini *et al.* (1991 ▶, 1992 ▶, 2001 ▶); Cappelli *et al.* (2008 ▶); Pittala *et al.* (2008 ▶).
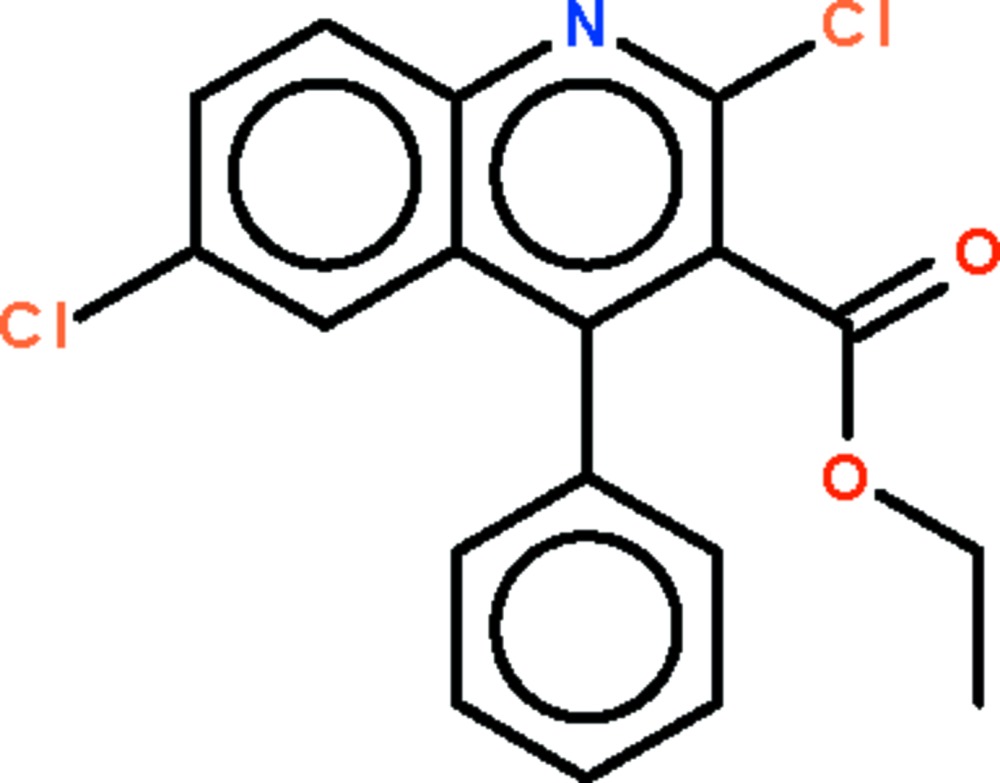


## Experimental

### 

#### Crystal data

C_18_H_13_Cl_2_NO_2_
*M* *_r_* = 346.19
Triclinic, 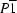

*a* = 8.3553 (3) Å
*b* = 10.1861 (5) Å
*c* = 10.6731 (6) Å
α = 110.537 (5)°
β = 101.421 (4)°
γ = 95.980 (4)°
*V* = 818.73 (7) Å^3^
*Z* = 2
Mo *K*α radiation
μ = 0.40 mm^−1^
*T* = 295 K
0.34 × 0.26 × 0.25 mm

#### Data collection

Bruker SMART CCD area-detector diffractometer
Absorption correction: multi-scan (*SADABS*; Sheldrick, 1996 ▶) *T*_min_ = 0.875, *T*_max_ = 0.906
18102 measured reflections
3700 independent reflections
2537 reflections with *I* > 2σ(*I*)
*R*_int_ = 0.031

#### Refinement

*R*[*F* ^2^ > 2σ(*F* ^2^)] = 0.046
*wR*(*F* ^2^) = 0.144
*S* = 1.02
3700 reflections
209 parameters
1 restraint
H-atom parameters constrained
Δρ_max_ = 0.41 e Å^−3^
Δρ_min_ = −0.36 e Å^−3^

### 

Data collection: *SMART* (Bruker, 2004 ▶); cell refinement: *SAINT* (Bruker, 2004 ▶); data reduction: *SAINT*; program(s) used to solve structure: *SHELXS97* (Sheldrick, 2008 ▶); program(s) used to refine structure: *SHELXL97* (Sheldrick, 2008 ▶); molecular graphics: *X-SEED* (Barbour, 2001 ▶); software used to prepare material for publication: *publCIF* (Westrip, 2009 ▶).

## Supplementary Material

Crystal structure: contains datablocks global, I. DOI: 10.1107/S1600536809045334/ci2954sup1.cif

Structure factors: contains datablocks I. DOI: 10.1107/S1600536809045334/ci2954Isup2.hkl

Additional supplementary materials:  crystallographic information; 3D view; checkCIF report

## Figures and Tables

**Fig. 1.**
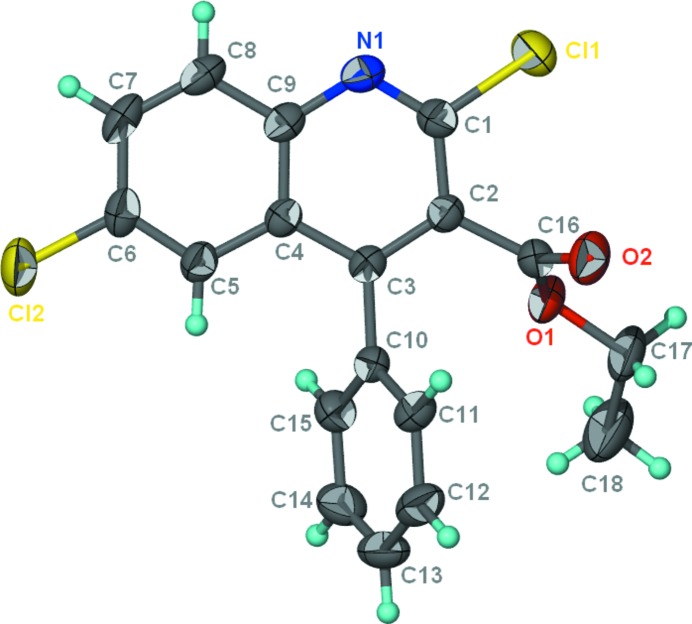
Displacement ellipsoid plot (Barbour, 2001) of C18H13Cl2NO2 at the 50% probability level. H atoms are drawn as spheres of arbitrary radius.

## supplementary crystallographic information

### Experimental

An excess of phosphorus oxychloride (0.9 ml, 10 mmol) and 6-chloro-1,2-dihydro-2-oxo-4-phenylquinoline-3-carboxylate (0.33 g, 1 mmol) were heated for 1 h. The mixture was then added to crushed ice. The solid that formed was collected and recrystallized from methanol.

### Refinement

C-bound H-atoms were placed in calculated positions (C-H = 0.93–0.97 Å) and were included in the refinement in the riding model approximation, with *U_iso_*(H) set to 1.2–1.5*U_eq_*(C). The C—C distance of the ethyl chain was tightly restrained to 1.500 (2) Å.

### Crystal data

**Table:**

C_18_H_13_Cl_2_NO_2_	*Z* = 2
*M**_r_* = 346.19	*F*(000) = 356
Triclinic, *P*1	*D*_x_ = 1.404 Mg m^−^^3^
Hall symbol: -P 1	Mo *K*α radiation, λ = 0.71073 Å
*a* = 8.3553 (3) Å	Cell parameters from 1235 reflections
*b* = 10.1861 (5) Å	θ = 1.7–21.3°
*c* = 10.6731 (6) Å	µ = 0.40 mm^−^^1^
α = 110.537 (5)°	*T* = 295 K
β = 101.421 (4)°	Block, colourless
γ = 95.980 (4)°	0.34 × 0.26 × 0.25 mm
*V* = 818.73 (7) Å^3^	

### Data collection

**Table:**

Bruker SMART CCD area-detector diffractometer	3700 independent reflections
Radiation source: fine-focus sealed tube	2537 reflections with *I* > 2σ(*I*)
graphite	*R*_int_ = 0.031
φ and ω scans	θ_max_ = 27.5°, θ_min_ = 3.4°
Absorption correction: multi-scan (*SADABS*; Sheldrick, 1996)	*h* = −10→10
*T*_min_ = 0.875, *T*_max_ = 0.906	*k* = −13→12
18102 measured reflections	*l* = −13→13

### Refinement

**Table:**

Refinement on *F*^2^	Primary atom site location: structure-invariant direct methods
Least-squares matrix: full	Secondary atom site location: difference Fourier map
*R*[*F*^2^ > 2σ(*F*^2^)] = 0.046	Hydrogen site location: inferred from neighbouring sites
*wR*(*F*^2^) = 0.144	H-atom parameters constrained
*S* = 1.02	*w* = 1/[σ^2^(*F*_o_^2^) + (0.07*P*)^2^ + 0.2927*P*] where *P* = (*F*_o_^2^ + 2*F*_c_^2^)/3
3700 reflections	(Δ/σ)_max_ = 0.001
209 parameters	Δρ_max_ = 0.41 e Å^−^^3^
1 restraint	Δρ_min_ = −0.35 e Å^−^^3^

### Fractional atomic coordinates and isotropic or equivalent isotropic displacement parameters (Å^2^)

**Table:**

	*x*	*y*	*z*	*U*_iso_*/*U*_eq_	
Cl1	0.38082 (9)	0.27696 (7)	0.20007 (7)	0.0656 (2)	
Cl2	0.09541 (10)	0.80809 (9)	0.87358 (6)	0.0737 (3)	
N1	0.2968 (2)	0.38235 (19)	0.42803 (19)	0.0464 (4)	
O1	0.4425 (2)	0.66274 (18)	0.20689 (15)	0.0522 (4)	
O2	0.2357 (2)	0.48698 (19)	0.05569 (16)	0.0651 (5)	
C1	0.3141 (3)	0.4086 (2)	0.3210 (2)	0.0432 (5)	
C2	0.2874 (3)	0.5342 (2)	0.2978 (2)	0.0394 (5)	
C3	0.2357 (3)	0.6384 (2)	0.3963 (2)	0.0372 (5)	
C4	0.2145 (3)	0.6144 (2)	0.5166 (2)	0.0381 (5)	
C5	0.1637 (3)	0.7137 (2)	0.6250 (2)	0.0436 (5)	
H5	0.1397	0.7989	0.6195	0.052*	
C6	0.1503 (3)	0.6832 (3)	0.7373 (2)	0.0479 (5)	
C7	0.1808 (3)	0.5550 (3)	0.7485 (2)	0.0534 (6)	
H7	0.1695	0.5368	0.8261	0.064*	
C8	0.2271 (3)	0.4575 (3)	0.6448 (2)	0.0509 (6)	
H8	0.2459	0.3714	0.6512	0.061*	
C9	0.2472 (3)	0.4845 (2)	0.5278 (2)	0.0421 (5)	
C10	0.2017 (3)	0.7710 (2)	0.3758 (2)	0.0399 (5)	
C11	0.0839 (3)	0.7647 (3)	0.2608 (2)	0.0479 (5)	
H11	0.0235	0.6769	0.1963	0.057*	
C12	0.0565 (3)	0.8896 (3)	0.2421 (3)	0.0607 (7)	
H12	−0.0235	0.8855	0.1659	0.073*	
C13	0.1478 (4)	1.0192 (3)	0.3363 (3)	0.0650 (7)	
H13	0.1301	1.1024	0.3227	0.078*	
C14	0.2633 (4)	1.0268 (3)	0.4490 (3)	0.0641 (7)	
H14	0.3240	1.1151	0.5123	0.077*	
C15	0.2910 (3)	0.9030 (2)	0.4697 (2)	0.0506 (6)	
H15	0.3701	0.9088	0.5472	0.061*	
C16	0.3163 (3)	0.5558 (2)	0.1716 (2)	0.0443 (5)	
C17	0.4740 (4)	0.7060 (3)	0.0971 (3)	0.0749 (9)	
H17A	0.3713	0.6852	0.0267	0.090*	
H17B	0.5522	0.6533	0.0547	0.090*	
C18	0.5432 (6)	0.8615 (3)	0.1550 (4)	0.1137 (15)	
H18A	0.5572	0.8916	0.0815	0.170*	
H18B	0.6490	0.8806	0.2195	0.170*	
H18C	0.4683	0.9129	0.2016	0.170*	

### Atomic displacement parameters (Å^2^)

**Table:**

	*U*^11^	*U*^22^	*U*^33^	*U*^12^	*U*^13^	*U*^23^
Cl1	0.0792 (5)	0.0546 (4)	0.0702 (4)	0.0224 (3)	0.0354 (4)	0.0209 (3)
Cl2	0.0878 (5)	0.1001 (6)	0.0420 (3)	0.0331 (4)	0.0288 (3)	0.0264 (3)
N1	0.0512 (11)	0.0404 (10)	0.0507 (11)	0.0064 (8)	0.0109 (9)	0.0226 (8)
O1	0.0616 (10)	0.0593 (10)	0.0393 (8)	0.0039 (8)	0.0178 (7)	0.0223 (7)
O2	0.0851 (13)	0.0648 (11)	0.0359 (9)	0.0015 (10)	0.0110 (9)	0.0135 (8)
C1	0.0424 (12)	0.0393 (11)	0.0472 (12)	0.0081 (9)	0.0125 (9)	0.0148 (9)
C2	0.0419 (12)	0.0405 (11)	0.0377 (10)	0.0071 (9)	0.0108 (9)	0.0170 (8)
C3	0.0372 (11)	0.0390 (11)	0.0361 (10)	0.0051 (9)	0.0072 (8)	0.0167 (8)
C4	0.0366 (11)	0.0431 (11)	0.0348 (10)	0.0053 (9)	0.0062 (8)	0.0171 (8)
C5	0.0448 (12)	0.0528 (13)	0.0375 (11)	0.0119 (10)	0.0111 (9)	0.0210 (9)
C6	0.0428 (12)	0.0664 (15)	0.0338 (11)	0.0074 (11)	0.0098 (9)	0.0189 (10)
C7	0.0545 (14)	0.0694 (16)	0.0382 (12)	−0.0051 (12)	0.0054 (10)	0.0309 (11)
C8	0.0577 (15)	0.0511 (13)	0.0479 (13)	0.0015 (11)	0.0063 (11)	0.0298 (11)
C9	0.0406 (12)	0.0441 (11)	0.0423 (11)	0.0017 (9)	0.0065 (9)	0.0212 (9)
C10	0.0461 (12)	0.0414 (11)	0.0398 (11)	0.0128 (9)	0.0176 (9)	0.0193 (9)
C11	0.0505 (14)	0.0519 (13)	0.0484 (12)	0.0093 (10)	0.0132 (10)	0.0272 (10)
C12	0.0577 (16)	0.0768 (19)	0.0698 (16)	0.0248 (14)	0.0205 (13)	0.0486 (15)
C13	0.0747 (19)	0.0517 (15)	0.091 (2)	0.0255 (14)	0.0355 (16)	0.0417 (15)
C14	0.0769 (19)	0.0414 (13)	0.0749 (18)	0.0124 (13)	0.0264 (15)	0.0191 (12)
C15	0.0595 (15)	0.0447 (12)	0.0461 (12)	0.0109 (11)	0.0142 (11)	0.0148 (10)
C16	0.0535 (14)	0.0451 (12)	0.0397 (12)	0.0166 (11)	0.0175 (10)	0.0173 (9)
C17	0.115 (2)	0.0674 (17)	0.0481 (14)	−0.0004 (16)	0.0374 (15)	0.0240 (13)
C18	0.189 (4)	0.082 (2)	0.075 (2)	−0.008 (3)	0.052 (3)	0.0349 (18)

### Geometric parameters (Å, °)

**Table:**

Cl1—C1	1.732 (2)	C8—C9	1.407 (3)
Cl2—C6	1.742 (2)	C8—H8	0.93
N1—C1	1.292 (3)	C10—C15	1.385 (3)
N1—C9	1.369 (3)	C10—C11	1.390 (3)
O1—C16	1.325 (3)	C11—C12	1.389 (3)
O1—C17	1.448 (3)	C11—H11	0.93
O2—C16	1.199 (3)	C12—C13	1.375 (4)
C1—C2	1.417 (3)	C12—H12	0.93
C2—C3	1.382 (3)	C13—C14	1.358 (4)
C2—C16	1.499 (3)	C13—H13	0.93
C3—C4	1.429 (3)	C14—C15	1.389 (4)
C3—C10	1.488 (3)	C14—H14	0.93
C4—C5	1.416 (3)	C15—H15	0.93
C4—C9	1.419 (3)	C17—C18	1.485 (2)
C5—C6	1.361 (3)	C17—H17A	0.97
C5—H5	0.93	C17—H17B	0.97
C6—C7	1.397 (4)	C18—H18A	0.96
C7—C8	1.356 (4)	C18—H18B	0.96
C7—H7	0.93	C18—H18C	0.96
			
C1—N1—C9	117.23 (18)	C11—C10—C3	120.90 (19)
C16—O1—C17	116.36 (18)	C12—C11—C10	120.0 (2)
N1—C1—C2	125.8 (2)	C12—C11—H11	120.0
N1—C1—Cl1	115.05 (16)	C10—C11—H11	120.0
C2—C1—Cl1	119.09 (17)	C13—C12—C11	120.0 (2)
C3—C2—C1	118.10 (19)	C13—C12—H12	120.0
C3—C2—C16	120.40 (18)	C11—C12—H12	120.0
C1—C2—C16	121.50 (19)	C14—C13—C12	120.6 (2)
C2—C3—C4	118.21 (18)	C14—C13—H13	119.7
C2—C3—C10	120.49 (18)	C12—C13—H13	119.7
C4—C3—C10	121.30 (18)	C13—C14—C15	120.1 (3)
C5—C4—C9	118.57 (19)	C13—C14—H14	120.0
C5—C4—C3	123.39 (19)	C15—C14—H14	120.0
C9—C4—C3	118.04 (19)	C10—C15—C14	120.5 (2)
C6—C5—C4	119.4 (2)	C10—C15—H15	119.8
C6—C5—H5	120.3	C14—C15—H15	119.8
C4—C5—H5	120.3	O2—C16—O1	125.1 (2)
C5—C6—C7	122.4 (2)	O2—C16—C2	124.8 (2)
C5—C6—Cl2	119.40 (19)	O1—C16—C2	110.14 (18)
C7—C6—Cl2	118.20 (17)	O1—C17—C18	109.4 (2)
C8—C7—C6	119.2 (2)	O1—C17—H17A	109.8
C8—C7—H7	120.4	C18—C17—H17A	109.8
C6—C7—H7	120.4	O1—C17—H17B	109.8
C7—C8—C9	121.1 (2)	C18—C17—H17B	109.8
C7—C8—H8	119.5	H17A—C17—H17B	108.2
C9—C8—H8	119.5	C17—C18—H18A	109.5
N1—C9—C8	118.0 (2)	C17—C18—H18B	109.5
N1—C9—C4	122.57 (19)	H18A—C18—H18B	109.5
C8—C9—C4	119.4 (2)	C17—C18—H18C	109.5
C15—C10—C11	118.9 (2)	H18A—C18—H18C	109.5
C15—C10—C3	120.20 (19)	H18B—C18—H18C	109.5
			
C9—N1—C1—C2	0.5 (3)	C7—C8—C9—C4	−1.6 (3)
C9—N1—C1—Cl1	178.84 (15)	C5—C4—C9—N1	179.98 (19)
N1—C1—C2—C3	−1.1 (3)	C3—C4—C9—N1	−0.4 (3)
Cl1—C1—C2—C3	−179.37 (16)	C5—C4—C9—C8	0.5 (3)
N1—C1—C2—C16	178.0 (2)	C3—C4—C9—C8	−179.88 (19)
Cl1—C1—C2—C16	−0.3 (3)	C2—C3—C10—C15	−119.8 (2)
C1—C2—C3—C4	0.8 (3)	C4—C3—C10—C15	60.9 (3)
C16—C2—C3—C4	−178.25 (19)	C2—C3—C10—C11	58.2 (3)
C1—C2—C3—C10	−178.5 (2)	C4—C3—C10—C11	−121.1 (2)
C16—C2—C3—C10	2.4 (3)	C15—C10—C11—C12	−0.5 (3)
C2—C3—C4—C5	179.40 (19)	C3—C10—C11—C12	−178.6 (2)
C10—C3—C4—C5	−1.3 (3)	C10—C11—C12—C13	1.0 (4)
C2—C3—C4—C9	−0.2 (3)	C11—C12—C13—C14	−0.9 (4)
C10—C3—C4—C9	179.14 (19)	C12—C13—C14—C15	0.3 (4)
C9—C4—C5—C6	1.1 (3)	C11—C10—C15—C14	−0.1 (4)
C3—C4—C5—C6	−178.5 (2)	C3—C10—C15—C14	178.0 (2)
C4—C5—C6—C7	−1.7 (3)	C13—C14—C15—C10	0.2 (4)
C4—C5—C6—Cl2	177.61 (16)	C17—O1—C16—O2	5.6 (3)
C5—C6—C7—C8	0.7 (4)	C17—O1—C16—C2	−173.7 (2)
Cl2—C6—C7—C8	−178.63 (18)	C3—C2—C16—O2	−114.0 (3)
C6—C7—C8—C9	1.0 (4)	C1—C2—C16—O2	66.9 (3)
C1—N1—C9—C8	179.8 (2)	C3—C2—C16—O1	65.3 (3)
C1—N1—C9—C4	0.3 (3)	C1—C2—C16—O1	−113.8 (2)
C7—C8—C9—N1	179.0 (2)	C16—O1—C17—C18	148.7 (3)
